# 46. Subglottic stenosis in a patient with granulomatosis with polyangiitis recently treated with rituximab

**DOI:** 10.1093/rap/rky034.009

**Published:** 2018-09-20

**Authors:** Asim Khan, Myles Lewis, Gavin Thomas

**Affiliations:** 1 Rheumatology, Barts Health NHS Foundation Trust, London, UNITED KINGDOM; 2 Respiratory Medicine, Barts Health NHS Foundation Trust, London, UNITED KINGDOM

**Introduction:** We present a 24-year-old Caucasian female recently diagnosed with granulomatosis with polyangiitis. Diagnosis was based on a presentation of ENT and respiratory symptoms, bilateral cavitating lung lesions, constitutional symptoms, raised inflammatory markers and positive PR3 antibody. Nasal biopsy demonstrated inflammatory ulceration and bronchoalveolar lavage revealed neutrophilic inflammation with *Pseudomonas aeruginosa* grown on culture. She was treated with high-dose corticosteroids, plasma exchange therapy, rituximab and antibiotic therapy. Six weeks later she was admitted from the emergency respiratory clinic with biphasic stridor, productive cough, polyphonic wheeze, tachypnoea, fevers and raised inflammatory markers. This case reflects the aggressiveness of small-vessel vasculitis in this patient despite recent immunosuppression. It also highlights the development of airway complications in vasculitis which is uncommon and under-appreciated. Finally, management was complex in light of *Pseudomonas aeruginosa* colonisation, and risks of infertility with cyclophosphamide required addressing.

**Case description:** A 24-year-old Caucasian waitress presented to the emergency department with productive cough, haemoptysis, fevers, night sweats and fatigue. Further clinical assessment revealed a history of chronic sinusitis, nasal crusting and epistaxis. On examination she was pyrexic, hypoxic, tachypnoeic, tachycardic with coarse crackles on auscultation of the right lung base. There was evidence of nasal bridge collapse too. Clinical assessment of the other major organ systems including the neurological, abdominal and cardiovascular systems was unremarkable. Her co-morbidities included well-controlled asthma for which she had two non-ITU hospital admissions as an adolescent. Her current medication consisted of seretide and salbutamol inhalers, and beclomethasone nasal spray. She was a never-smoker, did not use illicit drugs and alcohol intake was low. Blood tests demonstrated raised inflammatory markers (CRP 334 mg/L, ESR 130 mm/hour) and leucocytosis (20.4 x 10^9^/L) with neutrophilia (17.1 x 10^9^/L) in the absence of eosinophilia. Arterial blood gas confirmed type one respiratory failure. Chest radiograph revealed right middle zone cavitation. She was treated for pneumonia with co-amoxiclav and tamiflu, and high-flow oxygen with little improvement; she was subsequently transferred to critical care for respiratory support. A CT-chest confirmed bilateral multiple cavitating lesions and tracheobronchitis. Virology screen, sputum and blood cultures were unremarkable. Autoimmune serology was positive for PR3-ANCA (40 kUnits/L). Renal function, urine PCR and urine cytology were unremarkable. Troponin T was 55 ng/L; the patient had a normal ECG and echocardiogram. She was therefore diagnosed with granulomatosis with polyangiitis and commenced on 1 gram intravenous methylprednisolone for three days followed by a weaning regimen of 60 mg prednisolone. Despite this the patient had ongoing haemoptysis with a fall in haemoglobin so therefore received seven cycles of plasma exchange. Nasal mucosa biopsy revealed non-specific inflammatory ulceration with underlying lymphocytic infiltrate in the absence of eosinophils. A bronchoscopy confirmed bronchitis; subsequent bronchoalveolar lavage grew *Pseudomonas aeruginosa* on culture with neutrophilic inflammation on microscopy. Malignancy and TB were excluded. Because of her age and patient choice, she was treated with two doses of one-gram rituximab which was administered after completion of plasma exchange therapy. Antibiotics were also escalated to tazocin to treat *Pseudomonas aeruginosa*. She was discharged following four weeks as an inpatient. Six weeks later the patient was admitted from the emergency respiratory clinic with biphasic stridor, productive cough, tachypnoea, polyphonic wheeze and fevers. Her nasal bridge collapse had worsened too. An urgent fine nasal endoscopy demonstrated subglottic and distal airway purulent secretions with appearances highly suggestive of subglottic stenosis. She was treated with adrenaline and salbutamol nebulisers, oxygen therapy and high-dose intravenous dexamethasone (8 mg BD) resulting in resolution of stridor. Blood tests confirmed raised CRP (73 mg/L). Repeat PR3-ANCA titre was 23 kUnits/L despite recent rituximab, corticosteroids and plasma exchange. A chest radiograph again showed right lung opacification though improving. Sputum culture grew *Pseudomonas aeruginosa*; tazocin was therefore recommenced. Urinalysis, urine cytology and urine PCR were again unremarkable. IgA (0.56 g/L) and IgG (4.9 g/L) were both reduced. Lymphocyte subset analysis confirmed a CD19 < 1 x 10^6^/L. A CT-sinus demonstrated ongoing aggressive sinonasal inflammation. CT-neck revealed right-sided distortion at the subglottic angle. A repeat CT-thorax demonstrated improving appearances of the cavitating lung lesions. She was subsequently discussed in a Rheumatology-Nephrology-Respiratory multidisciplinary meeting; in light of ongoing vasculitic activity despite recent immunosuppression a collective decision was made to commence cyclophosphamide. However, in light of *Pseudomonas aeruginosa* colonisation the patient would also receive antibiotic therapy whilst on cyclophosphamide. Ten days following admission a microlaryngoscopy and bronchoscopy was performed which revealed resolution of inflammation and no evidence of airway stenosis. She was discussed with our fertility clinic and subsequently initiated on monthly subcutaneous leuprorelin for ovarian protection. Intravenous cyclophosphamide 750 mg was subsequently commenced via the CYCLOPS regimen. Dexamethasone was stopped and converted to high-dose prednisolone - this was weaned as per CYCLOPS protocol. Following discharge, tazocin was switched to colistin nebuliser treatment.

**Discussion:** Subglottic stenosis is an uncommon life-threatening manifestation of granulomatosis with polyangiitis (GPA) and can occur both at diagnosis and subsequent to it. It presents with stridor, dyspnoea, cough and hoarseness. Diagnosis is achieved by direct visualisation via fine nasal endoscopy; imaging can also aid the diagnosis. Treatment is challenging though consists of systemic or local corticosteroids and corticosteroid-sparing agents including cyclophosphamide and rituximab. Surgical treatment includes balloon dilatation and stenting. Our case is notable for various reasons. Firstly the patient was diagnosed with GPA at a young age whereas GPA typically affects patients from the fifth decade onwards. In addition her disease primarily affected the ENT and respiratory systems with absence of renal involvement. Because of her age the use of cyclophosphamide as the initial induction agent had to be carefully balanced. Following discussions with our patient regarding the advantages and disadvantages of cyclophosphamide and a discussion regarding alternatives such as rituximab, she chose rituximab. We also gave plasma exchange therapy in light of significant haemoptysis-induced anaemia and respiratory failure. Despite powerful immunosuppression within six weeks our patient presented with an upper airway emergency secondary to active vasculitis. Of interest other reports of tracheobronchial involvement in GPA also have largely involved young females. Moreover the current literature suggests that rituximab rather than cyclophosphamide is more likely to resolve upper airway inflammation whereas our patient presented with airway complications following recent rituximab therapy. Our case was further complicated by the presence of *Pseudomonas aeruginosa* colonisation which required sustained treatment whilst the patient received cyclophosphamide to reduce the risk of respiratory tract infection.

**Key Learning Points:** This case highlights the importance of a multidisciplinary approach in achieving the best outcome for our patient. We worked alongside other specialities including respiratory medicine, nephrology, ENT and obstetrics to provide optimum safe care. Furthermore, at the onset of diagnosis we had a discussion with the patient regarding treatment options including cyclophosphamide and rituximab for induction of remission. Patient involvement where appropriate in treatment choice is important and ensures that the patient is equally involved in the shared decision making process. Our patient had an aggressive vasculitis despite initial treatment with corticosteroids, rituximab and plasma exchange. This was further complicated by the presence of *Pseudomonas aeruginosa* colonisation. Imaging and PR3-ANCA titres were particularly helpful in establishing ongoing vasculitic activity and helping differentiate it from infection-induced inflammation. This case would also help initiate discussions regarding the management of ANCA-positive vasculitis in patients recently treated who have subsequently relapsed. Subglottic stenosis is an under-recognised manifestation of granulomatosis with polyangiitis. This case helps raise awareness, it also provides a platform for discussion regarding the challenges in treating it given that it may not always respond to conventional immunosuppression.

**Disclosure: A. Khan:** None. **M. Lewis:** None. **G. Thomas:** None.

